# Impact of Tumour Biology on Outcomes of Radical Therapy for Hepatocellular Carcinoma Oligo-Recurrence after Liver Transplantation

**DOI:** 10.3390/jcm11154389

**Published:** 2022-07-28

**Authors:** Kin-Pan Au, James Yan-Yue Fung, Wing-Chiu Dai, Albert Chi-Yan Chan, Chung-Mau Lo, Kenneth Siu-Ho Chok

**Affiliations:** 1 Department of Surgery, The University of Hong Kong, Hong Kong, China; keithkpau@gmail.com (K.-P.A.); jeffdai02@yahoo.com.hk (W.-C.D.); acchan@hku.hk (A.C.-Y.C.); chungmlo@hku.hk (C.-M.L.); 2 Department of Medicine, The University of Hong Kong, Hong Kong, China; sicklehut@gmail.com

**Keywords:** liver transplant, hepatocellular carcinoma, oligo-recurrence

## Abstract

It is uncertain whether tumour biology affects radical treatment for post-transplant hepatocellular carcinoma (HCC) oligo-recurrence, i.e. recurrence limited in numbers and locations amendable to radical therapy. We conducted a retrospective study on 144 patients with post-transplant HCC recurrence. Early recurrence within one year after transplant (HR 2.53, 95% CI 1.65–3.88, *p* < 0.001), liver recurrence (HR 1.74, 95% CI 1.12–2.68, *p* = 0.01) and AFP > 200 ng/mL upon recurrence (HR 1.62, 95% CI 1.04–2.52, *p* = 0.03) predicted mortality following recurrence. In patients with early recurrence and liver recurrence, radical treatment was associated with improved post-recurrence survival (early recurrence: median 18.2 ± 1.5 vs. 9.2 ± 1.5 months, *p* < 0.001; liver recurrence: median 28.0 ± 4.5 vs. 11.6 ± 2.0, *p* < 0.001). In patients with AFP > 200 ng/mL, improvement in survival did not reach statistical significance (median 18.2 ± 6.5 vs. 8.8 ± 2.2 months, *p* = 0.13). Survival benefits associated with radical therapy were reduced in early recurrence (13.6 vs. 9.0 months) and recurrence with high AFP (15.4 vs. 9.3 months) but were similar among patients with and without liver recurrence (16.9 vs. 16.4 months). They were also diminished in patients with multiple biological risk factors (0 risk factor: 29.0 months; 1 risk factor: 19.7 months; 2–3 risk factors: 3.4 months): The survival benefit following radical therapy was superior in patients with favourable biological recurrence but was also observed in patients with poor tumour biology. Treatment decisions should be individualised considering the oncological benefits, quality of life gain and procedural morbidity.

## 1. Introduction

Liver transplantation is the ideal treatment for early-stage hepatocellular carcinoma (HCC) in cirrhotic liver [1,2]. One-fifth of liver transplantations worldwide are now performed for HCC [3,4,5]. Stringent selection criteria have been adopted to optimise the outcomes [6,7]. Various prognostic models have also been developed and recurrence risk has been accurately predicted based on clinicopathological parameters [8,9,10]. Nevertheless, recurrence occurs in 20% of recipients transplanted for HCC [11,12]. The prognosis of post-transplant recurrence is generally poor. Recurrent tumours often progress unrestrainedly under suppressed host immunity. Palliation has been the mainstay treatment for these patients; however, improved immunosuppression and anti-tumour therapy have enhanced systemic control [13,14,15,16]. Thus, there has been a paradigm shift in the management of recurrent disease with limited numbers and location, i.e., oligo-recurrence [17], and patients are treated with a radical approach combining systemic and local therapy. Post-transplant recurrence represents metastatic disease from the native liver. Radical treatment for localised recurrence is only meaningful when systemic control is effective. The radical approach has been shown to confer survival benefits to patients with post-transplant oligo-recurrence [18].

However, it is uncertain which patients will enjoy improved survival or suffer early progression following radical therapy. Radical treatment in a transplant recipient is also a major undertaking. The immunocompromised hosts are susceptible to infective morbidities [19]. The surgical treatment of graft recurrence is particularly challenging with hostile adhesions and risk of damaging vital hilar structures [20,21,22]. To justify the risk, patient selection is essential to ensure favourable treatment outcomes. Disease volume is not the sole determinant of prognosis [18,23,24]. The biological behaviour of the recurrent tumour also affects mortality following HCC recurrence. Very little has been reported regarding how these biological factors affect patient selection for radical therapy. The current study was proposed to address the clinical question as to whether treating oligo-recurrence of poor tumour biology is worthwhile. We aimed to identify the biological risk factors of poor survival and to examine their impact on the treatment outcomes of post-transplant HCC oligo-recurrence.

## 2. Materials and Methods

A retrospective study was conducted at Queen Mary Hospital, the University of Hong Kong, a tertiary referral centre and the only liver transplant centre in Hong Kong. Patients transplanted for HCC attended outpatient follow-up every 3 months, during which clinical examination and blood tests for liver function and alpha-fetoprotein (AFP) were performed. A contrast-enhanced computed tomography (CT) scan of the thorax and abdomen was performed at 6-month intervals. HCC recurrence was diagnosed primarily on radiological grounds. All consecutive patients with proven HCC in the explant and diagnosed with HCC recurrence between January 2000 and March 2020 were included in this study. No donor organs were obtained from executed prisoners or other institutionalised persons.

The management of post-transplant HCC recurrence has been described elsewhere [25]. Briefly, upon recurrence, immunosuppression was tapered to the lowest effective dose. Consideration was given to an mTOR-based regime, with or without combination with reduced dose calcineurin inhibitor (tacrolimus with trough level < 5 ug/L). Staging was performed using a contrast CT) scan of the thorax and abdomen, and with a bone scan when the patient developed symptoms suggestive of bone metastasis. Dual-tracer positron emission tomography-computed tomography (PET-CT) was offered as an option at cost. The treatment plan was formulated based on patient and disease status. Oligo-recurrence was defined as recurrence limited in numbers and locations so that they were amendable to radical treatment. Patients with oligo-recurrence were selected for radical treatments. The treatment decisions were discussed by a multidisciplinary tumour board comprised of transplant surgeons, hepatologists, intervention radiologists, radiation oncologists and medical oncologists.

Data were retrieved from a prospectively maintained database, which included information regarding the transplant (tumour number and size, explant pathology), recurrence (timing of recurrence, number, size and location of recurrence, and level of AFP) and treatment characteristics (immunosuppression and modality of treatment). Patients were divided into three groups according to the treatment they received: radical, palliative and supportive. Radical therapy was defined as surgical resection or ablation. Palliative therapy included any other anti-tumour therapy including systemic therapy, regional therapy, e.g., trans-arterial chemoembolisation (TACE) and radiotherapy. The supportive group received no anti-tumour therapy. The primary endpoint was post-recurrence survival. Categorical variables were compared using the chi-square test. Continuous variables were presented as the median and interquartile range. Parametric variables were compared using the Student’s t test and non-parametric variables were compared using the Mann–Whitney U test. Factors affecting post-recurrence survival were studied with univariate and multivariate Cox-regression analysis. The survival outcomes of radical treatment for oligo-recurrence were studied with respect to these factors with the Kaplan–Meier method. Data were analysed using the Statistical Package for the Social Sciences 26.0 (SPSS) for Windows (SPSS Inc., Chicago, IL, USA). Statistical significance was defined by *p*-value < 0.05. This study was approved by the Institutional Review Board (IRB) of the University of Hong Kong/Hospital Authority Hong Kong West Cluster (IRB Reference Number: UW 21-736).

## 3. Results

### 3.1. Patient Characteristics

One hundred and forty-four patients with post-liver transplant HCC recurrence were included in this study. The details of the transplant, recurrence and treatment data are described in the Supplementary Materials, Table S1 and in our previous publication [15]. There was a median of three recurrent tumours (IQR 1-8), measuring up to a median size of 2.0 cm (IQR 1.1–3.4 cm). Thirty-five percent (*n* = 50) of the patients were treated with radical therapy while 53% (*n* = 76) and 13% (*n* = 18) were given palliative treatment and supportive care, respectively. Patients who received radical therapy had fewer recurrent tumours (1 vs. 4, *p* < 0.001), smaller tumours (1.8 vs. 2.5 cm, *p* = 0.046) and fewer involved organs (IQR 1-1 vs. 1–2, *p* = 0.02) (Table 1). The radical treatment group survived significantly longer than the palliative group (median survival 30.9 ± 2.4 vs. 12.6 ± 1.9 months, *p* < 0.001). Radical therapy included 60 surgical resections (liver: 10; lung: 36) and 16 ablations (Supplementary Materials, Table S2). Missing data were less than 5% for all variables except explant data. This was because 59% (*n* = 85) of the patients received the transplant procedure in other centres, and some of their explant data was not available. The remaining 59 (41%) patients received liver transplantation in our centre. Within the study period, 382 transplantations were performed in our centre for HCC. The recurrence rate was 15.4%.

### 3.2. Predictors of Survival

The median survival was 16.5 ± 1.1 months after recurrence. Multivariate predictors of mortality included recurrence within one year after transplant (HR 2.53, 95% CI 1.65–3.88, *p* < 0.001), size of the largest recurrence (HR 1.12, 95% CI 1.02–1.23, *p* = 0.03), number of involved organs (HR 1.48, 95% CI 1.05–2.07, *p* = 0.03), presence of liver recurrence (HR 1.74, 95% CI 1.12–2.68, *p* = 0.01) and AFP >200 ng/mL upon recurrence (HR 1.62, 95% CI 1.04–2.52, *p* = 0.03) (Table 2). mTOR inhibitor (HR 0.334, 95% CI 0.202–0.551, *p* < 0.001) and radical treatment (HR 0.327, 95% CI 0.203–0.528, *p* < 0.001) predicted improved survival.

### 3.3. Biological Factors and Survival

Among the identified risk factors, the size of the recurrence and number of involved organs reflected disease volume. Early recurrence, the presence of liver recurrence and high AFP level were factors potentially related to tumour biology.

**Figure 1:**
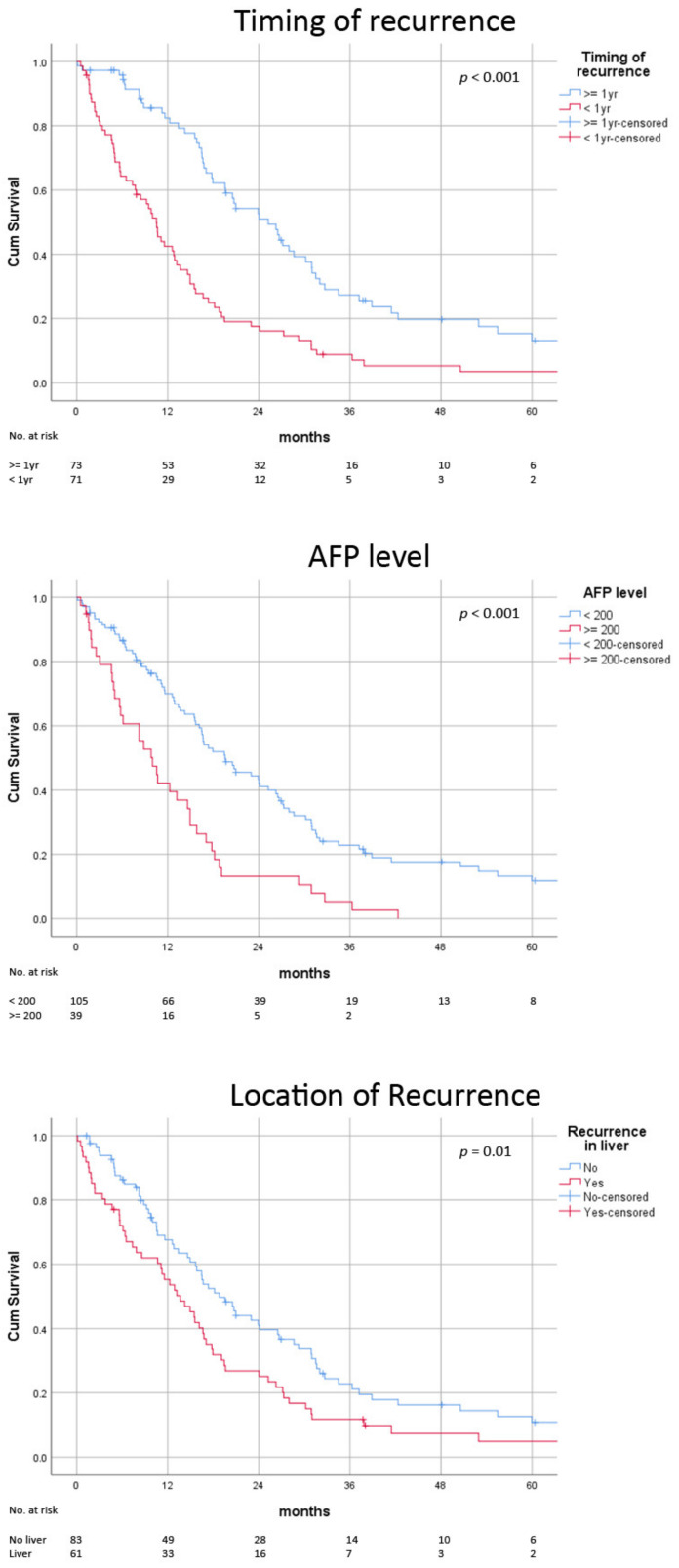
Post-recurrence survival stratified by biological risk factors.

The characteristics of patients with early and late recurrence were shown in Table 3. Early recurrence resulted from more advanced disease at transplant and more aggressive tumour behaviour. The early recurrence group had fewer patients within the criteria (Milan: 20% vs. 38%, *p* = 0.03; UCSF 24% vs. 43%, *p* = 0.02), a higher AFP level upon transplant (278 vs. 69 ng/mL, *p* = 0.01) and apparently more microvascular invasion in the explant (72% vs. 48%, *p* = 0.07). Early recurrence usually took place as a multiple relapse (number of recurrences 5 vs. 2, *p* = 0.001) in the lung (62% vs. 38%, *p* = 0.01). This contrasted with late recurrence, which more frequently occurred as a limited recurrence in other organs, e.g., peritoneum (4% vs. 14%, *p* = 0.047) and adrenal (4% vs. 12%, *p* = 0.08). The AFP level was also higher in early recurrence (85 vs. 7 ng/mL, *p* < 0.001). Early recurrence was seldom managed with radical intent (24% vs. 45%, *p* = 0.01) but was more frequently managed supportively (18% vs. 7%, *p* = 0.01), and was associated with inferior survival (median 10.5 ± 1.0 vs. 25.2 ± 3.3 months, *p* < 0.001) (Figure 1).

The high AFP group had some overlap with the early recurrence group (median time to recurrence 6 months vs. 14 months, *p* = 0.004) (Table 4). However, in contrast to early versus late recurrence, the high and low AFP group had similar disease status. Despite large difference in AFP (1128 vs. 7 ng/mL, *p* < 0.001), the number (3 vs. 3 *p* = 0.49) and size of recurrence (2.3 vs. 2.0 cm, *p* = 0.97) were similar. The only difference in disease volume was a marginally higher number of organs involved (IQR 1-2 vs. 1-1, *p* = 0.04). The tumour distribution was more similar (liver 44% vs. 42%, *p* = 0.86; lung 54% vs. 49%, *p* = 0.57, bone 18% vs. 15%, *p* = 0.69), apart from more distant lymph node metastases in the high AFP group (15% vs. 4%, *p* < 0.001). Despite comparable disease status, fewer patients with high AFP underwent radical therapy (18% vs. 41%, *p* = 0.01). The post-recurrence survival was limited compared to patients with low AFP (median 10.0 ± 1.8 vs. 19.5 ± 3.0 months, *p* < 0.001) (Figure 1).

Patients with recurrence in the liver graft had more advanced recurrence with larger tumours (median size 2.7 vs. 1.5 cm, *p* = 0.04) (Table 5). The number of affected organs was higher with liver recurrence (IQR 1-2 vs. 1-1, *p* < 0.001). Their survival was inferior to patients without liver recurrence (median 13.7 ± 2.2 vs. 18.8 ± 2.3 months, *p* = 0.01) (Figure 1).

### 3.4. Impact of Biological Factors on Radical Therapy

**Figure 2:**
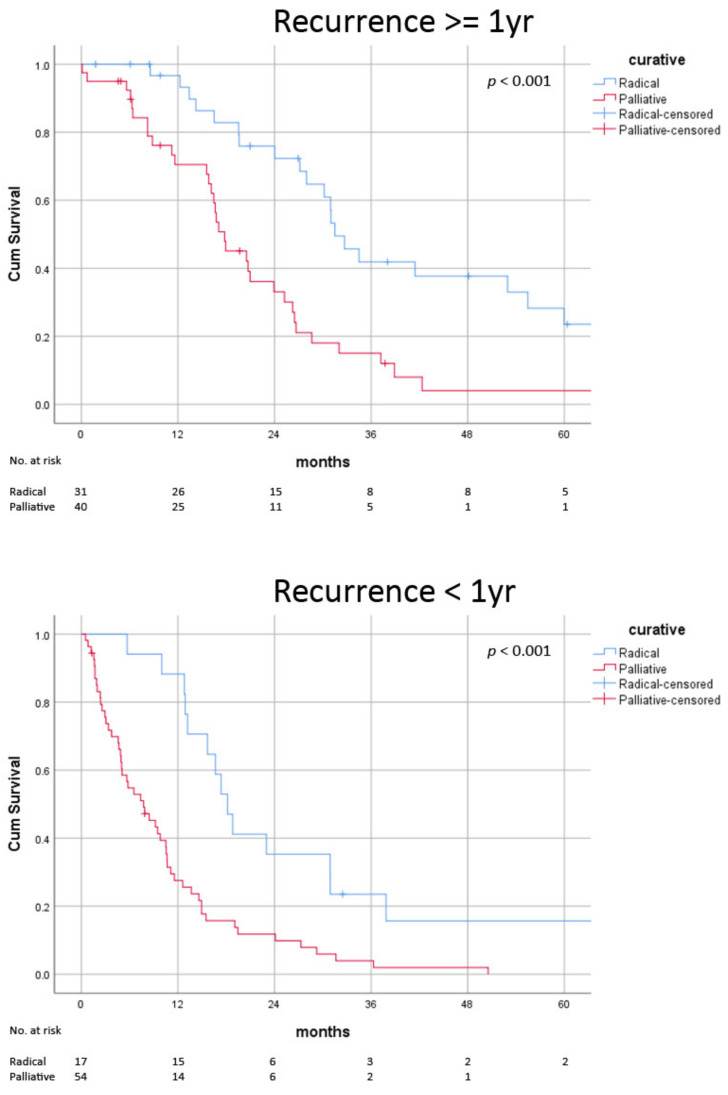
Post-recurrence survival of radical versus palliative treatment in late and early recurrence.

**Figure 3:**
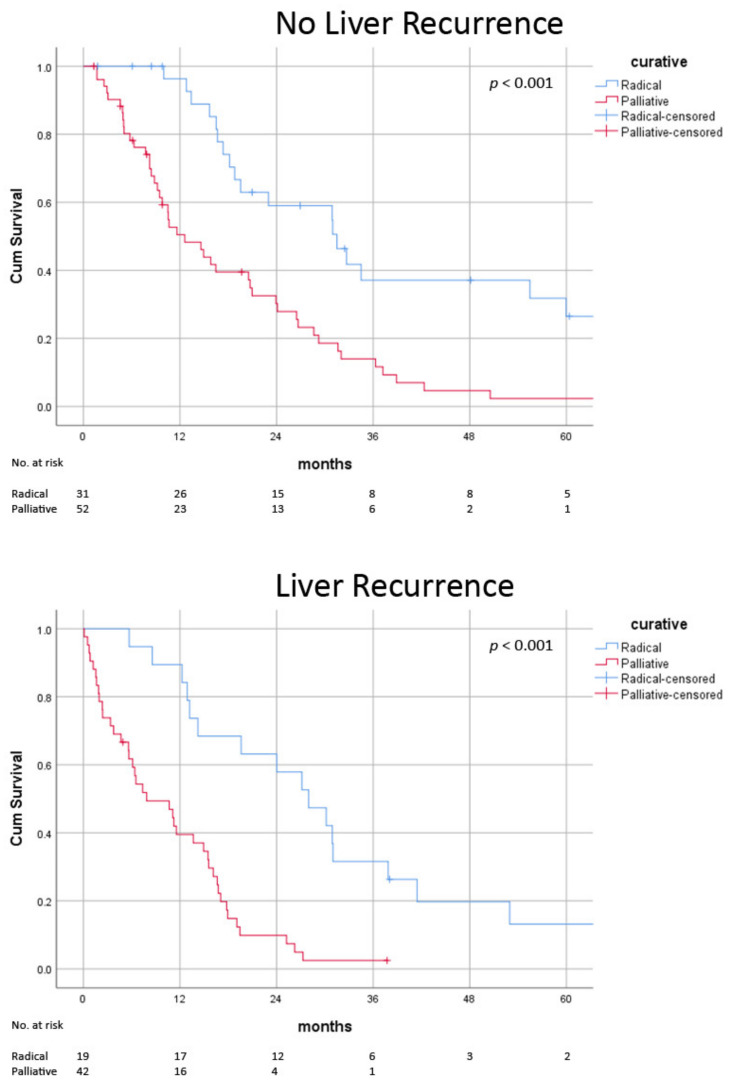
Post-recurrence survival of radical versus palliative treatment in absence and presence of liver recurrence.

**Figure 4:**
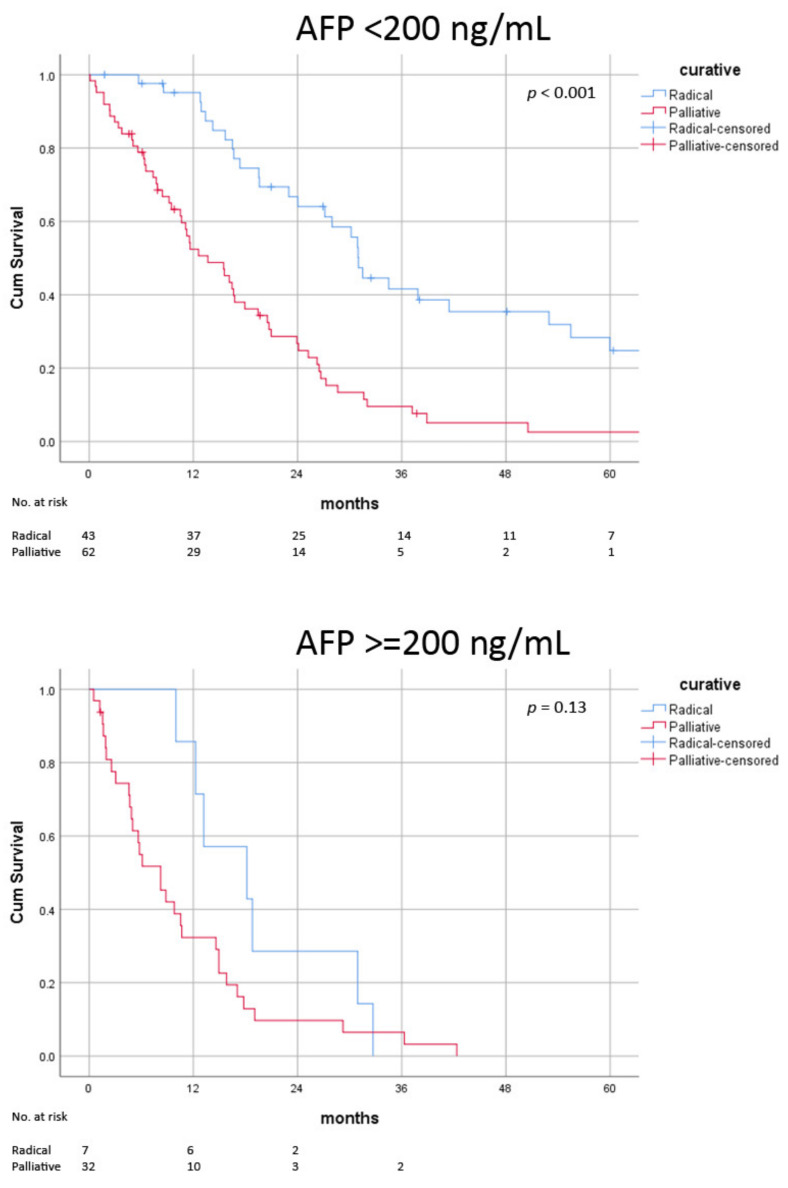
Post-recurrence survival of radical versus palliative treatment in low and high AFP level.

In the absence of poor risk factors, survival benefits were consistently observed in those receiving radical treatment (AFP < 200 ng/mL: median 31.0 ± 1.0 vs. 15.6 ± 2.7 months, *p* < 0.001; recurrence > 1 year: median 31.5 ± 2.2 vs. 17.9 ± 2.7 months, *p* < 0.001; no liver recurrence: median 31.5 ± 4.4 vs. 14.6 ± 3.3 months, *p* < 0.001) (Figure 2, Figure 3 and Figure 4) (Table 6). In patients with early recurrence (median 18.2 ± 1.5 vs. 9.2 ± 1.5 months, *p* < 0.001) and liver recurrence (median 28.0 ± 4.5 vs. 11.6 ± 2.0 months, *p* < 0.001), radical treatment was associated with improved survival (Figure 2 and Figure 3). In patients with high AFP at the time of recurrence, survival advantage with radical therapy was clinically apparent but did not reach statistical significance (median 18.2 ± 6.5 vs. 8.8 ± 2.2 months, *p* = 0.13) (Figure 4). The survival benefits with radical therapy were relatively diminished in patients with early recurrence (median 15.4 vs. 9.4 months) and high AFP (median 13.6 vs. 9.0 months) but were similar among patients with and without intra-hepatic recurrence (median 16.9 vs. 16.4 months).

**Figure 5:**
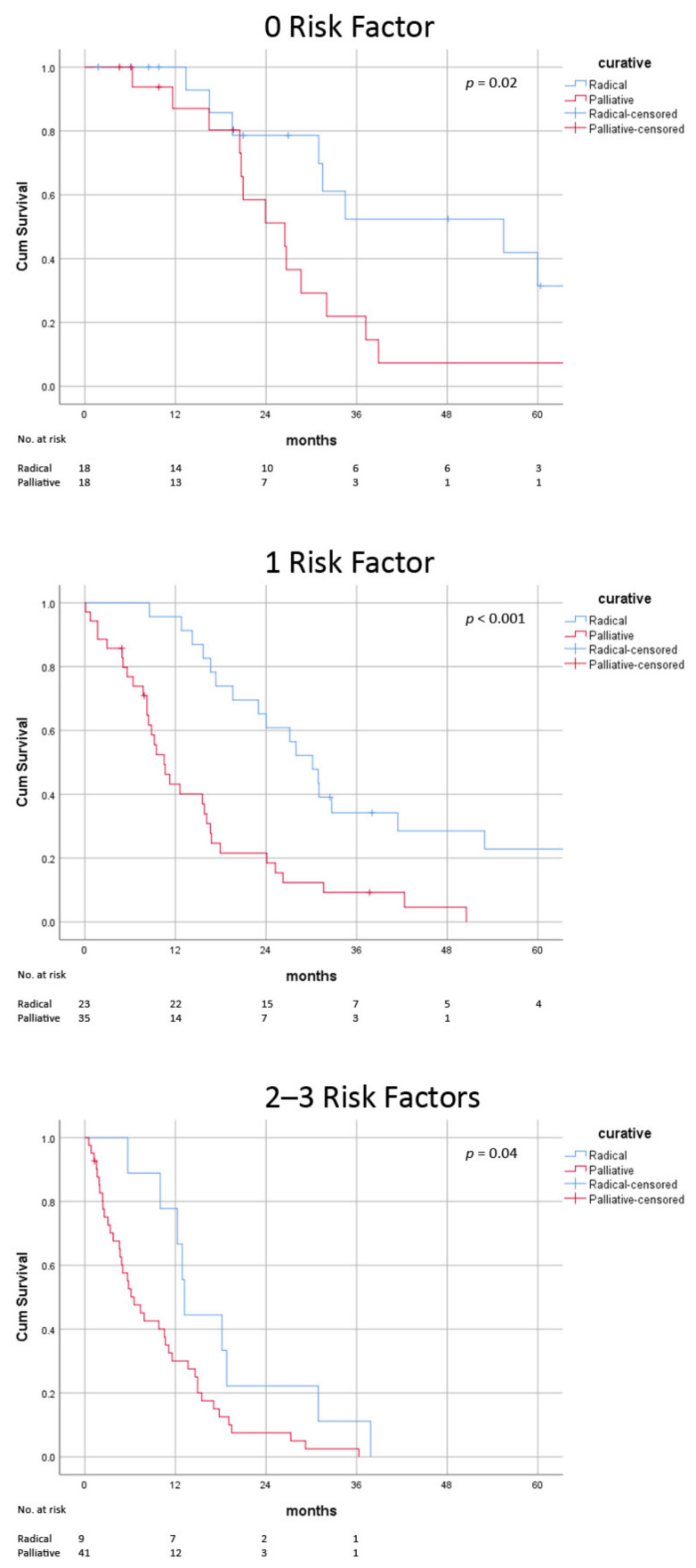
Post-recurrence survival of radical versus palliative treatment stratified by number of risk factors.

When the number of these risk factors were considered, improved survival was observed following radical treatment in patients with 0 (median 55.5 ± 18.5 vs. 26.5 ± 5.0 months, *p* = 0.02), 1 (median 30.2 ± 3.0 vs. 10.5 ± 1.7 months, *p* > 0.001) and 2–3 risk factors (median 13.2 ± 0.4 vs. 9.8 ± 2.3 months, *p* = 0.047) (Figure 5) (Table 6). The survival gain was relatively lower in patients with multiple risk factors (0: 29.0 months; 1: 19.7 months; 2–3: 3.4 months).

## 4. Discussion

We investigated how tumour biology affected the outcomes of radical therapy for post-transplant HCC recurrence. Early recurrence, high AFP level and liver recurrence were identified as biological factors that predict mortality. The benefits of radical treatment were superior in patients with good tumour biology, but survival improvement was also observed in patients with poor biological recurrence.

We observed that intra-hepatic recurrence predicted limited survival. Roayaie et al. and Bodzin et al. reported bone recurrence as a risk factor (Table 7) [23,24]. This discrepancy could be explained by the divergent recurrence status. We had strict surveillance protocol and recurrences were often detected early. Our patients had a median of 3 tumours (IQR 1-8) affecting 1 organ (IQR 1-1), with the largest tumour measuring 2 cm in size (IQR 1.1–3.4 cm), whereas in Bodzin et al.’s series, there were a median of more than 10 tumours affecting 2 organs, with the median size of the largest tumour measuring 4 cm [21]. Bone recurrence probably occurred in the later stage of the disease. We had fewer patients with skeletal metastasis (25% vs. 16%), which limited its importance in the survival analysis. The prognostic value of the location of recurrence could be further studied across different cohorts of patients.

Patients with liver recurrence had larger tumours (2.7 vs. 1.5 cm, *p* = 0.04). One possible explanation was that pulmonary metastasis of similar size is more readily detectable on axial imaging. Patients with liver recurrence had poorer prognosis. Post-transplant recurrence results from intra-operative tumour spillage or post-operative progression of undetected metastasis. While the implant is only engrafted after total hepatectomy, liver recurrence is more likely to take place via the latter mechanism and is probably associated with higher tumour load. We observed that liver recurrence predicted poorer survival, but the clinical significance was modest (median survival 18.8 vs. 13.7 months) (Table 4). Moreover, the survival benefit following radical treatment of liver recurrence was comparable to extrahepatic recurrence (no liver recurrence 16.9 vs. liver recurrence 16.4 months) (Figure 3). These results indicated that oligo-recurrence in liver was readily treatable with radical therapy. Resection or ablation should be considered in patients with limited recurrence in the liver graft.

In line with previous series, early recurrence and high AFP were predictors of mortality in our patients (Table 7) [23,24,26,27]. Early recurrence was usually multiple pulmonary metastasis while late recurrence was more often limited disease in other organs, e.g., peritoneum and adrenal gland. The distinctive patterns suggested that early and late relapse were caused via different mechanisms [28,29]. Early recurrence resulted from tumour spillage or the progression of occult metastasis and was more related to surgical manipulation and tumour factors [30]. As per our observation, early recurrence was associated with microvascular permeation. On the other hand, late recurrence could be the result of a resurgence of dormant seeds in the host, which explained the limited numbers and locations of the relapse. In post-resection late recurrence, this was related to hepatitis B viral load [31]. However, in the current cohort all patients received anti-viral and hepatitis B virus (HBV) titre was well suppressed. Immunosuppression could have played a role, but the exact mechanism of tumour resurgence remains poorly understood. Following early recurrence, fewer patients received radical therapy, probably due to more diffuse disease (24% vs. 45%, *p* = 0.01). Those receiving radical therapy survived longer (median 18.2 vs. 9.2 months, *p* < 0.001) (Figure 2). The survival difference between radical and palliative therapy was clinically significant (9.0 months), though it compared poorly to that in late recurrence (13.6 months).

Disease volume was similar in the high and low AFP group (tumour number 3 vs. 3 *p* = 0.49; size of recurrence 2.3 vs. 2.0 cm, *p* = 0.97), despite the huge difference in AFP (1128 vs. 7 ng/mL, *p* < 0.001). The AFP level in our patients probably reflected tumour biology more than disease volume. This was because recurrence was uniformly detected early. However, the limited availability of explant data precluded any statistical association (*n* = 10 available for degree of differentiation and *n* = 8 for microvascular permeation). Despite similar disease status, fewer patients with high AFP were managed with radical therapy (18% vs. 41%, *p* = 0.01), most notably surgery (13% vs. 38%, *p* = 0.004). More patients were given palliative treatment (64% vs. 49%, *p* = 0.004). Selection bias was probable because clinicians may avoid radical treatment for high AFP oligo-recurrence. The survival benefits associated with radical treatment in patients with high AFP were clinically apparent but did not have statistical significance (18.2 ± 6.5 vs. 8.8 *±* 2.2 months, *p* = 0.13) (Figure 4). This could have resulted from inadequate statistical power. There were only a few patients with high AFP receiving radical treatment (*n* = 7). The survival differences between the radical and palliative treatment were reduced (9.4 vs. 15.4 months) compared to those in patients with low AFP.

Radical treatment of oligo-recurrence offers oncological benefits [32]. However, clinicians have refrained from aggressive therapy for recurrence with unfavourable tumour biology. It is believed that outcome is dictated by tumour biology, irrespective of the therapeutic strategy. The results of the current study indicate that the survival benefit associated with radical therapy is superior in patients with favourable biological recurrence, but it is also observed in patients with poor tumour biology. Given limited disease volume and the opportunity to curation, it is logical and practical to pursue radical treatment for oligo-recurrence. When poor risk factors are present, the relative oncological disadvantage needs to be addressed. It is pertinent to note that not all oligo-recurrences are similar in terms of difficulty in achieving radical resection or ablation. A superficial liver oligo-recurrence might be more readily treatable than a deep-seated tumour situated close to a major pedicle. The improved quality of life with treatment, i.e., quality-adjusted life-year (QALY) gain may also warrant consideration. An individualised and informed decision is required for every patient, taking into account the potential benefits and drawback of each option. Treatment recommendations based solely on the behaviour of recurrence might disadvantage patients who might still benefit from radical therapy in spite of poor tumour biology.

The current study was limited by the retrospective design. Assessment was at risk of selection bias as the decision to implement radical treatment was not protocol-driven. There might be important bias as the treatment attitudes might have evolved during the lengthy study period (2000–2020). The definition for oligo-recurrence was arbitrary. The sample size was limited, which made matching a balanced cohort difficult. Incomplete data precluded effective analysis for explant characteristics. Nevertheless, we identified the poor prognostic factors in post-transplant oligo-recurrence and revealed that survival gains often persisted following radical therapy in poor biology recurrence. Radical therapy remains an option for oligo-recurrence with poor tumour biology. Treatment decisions can be personalised for each patient by considering the oncological benefits, quality of life gains and the procedural morbidities. Our findings could be validated in future studies using other patient cohorts. Future studies could also be directed to the optimal treatment strategy for poor biology recurrence. The potential role of systemic therapy, e.g., targeted therapy and immunotherapy as a selection tool for disease stabilisation before radical therapy can be explored.

## Figures and Tables

**Table 1:** Characteristics of patients who underwent radical and palliative therapy.

	Radical Therapy (*n* = 50) Median (IQR), *n* (%), or Median ± Standard Error		Palliative Therapy (*n* = 76) Median (IQR), *n* (%), or Median ± Standard Error		*p*-Value
Recurrence characteristics					
Age at recurrence	56	(50–62)	58	(50–63)	0.49
Time from transplant (months)	17	(11–41)	11	(5–17)	0.01 *
Number of recurrences	1	(1–3)	4	(2–8)	<0.001 *
Size of largest tumour (cm)	1.8	(0.9–2.9)	2.5	(1.2–3.7)	0.046 *
Number of organs involved	1	(1–1)	1	(1–2)	0.02 *
Site of recurrence					
Liver	19	(38%)	31	(41%)	0.75
Lung	26	(52%)	34	(45%)	0.42
Bone	2	(4%)	20	(26%)	0.01 *
Peritoneum	5	(10%)	7	(9%)	0.88
Adrenal	5	(10%)	6	(8%)	0.68
Lymph node	1	(2%)	7	(9%)	0.1
AFP upon recurrence (ng/mL)	7	(4–64)	40	(5–761)	0.01 *
Treatment characteristics					
Immunosuppression					
mTOR inhibitor	31	(62%)	48	(63%)	0.90
Calcineurin inhibitor	35	(70%)	60	(79%)	0.25
Radical therapy					
Surgery	45	(90%)			
Liver	9	(18%)			
Lung	27	(54%)			
Adrenal	6	(12%)			
Bone	1	(2%)			
Others	8	(16%)			
Ablation	16	(32%)			
RFA	15	(30%)			
Alcohol injection	1	(2%)			
Palliative therapy					
Local therapy					
HIFU	2	(4%)	1	(1%)	0.36
SBRT	7	(14%)	10	(13%)	0.89
Regional therapy					
TACE	15	(30%)	15	(20%)	0.19
SIRT	1	(2%)	0	(0%)	0.22
Systemic therapy	24	(48%)	51	(67%)	0.03 *
Targeted therapy	18	(36%)	49	(64%)	0.002 *
Chemotherapy	11	(22%)	14	(18%)	0.62
Immunotherapy	1	(2%)	2	(3%)	0.82
Median survival (months)		30.9 ± 2.4		12.6±1.9	<0.001 *

* signify statistical significance.

**Table 2:** Univariate and multivariate predictors of post-recurrence survival.

	Univariate			Multivariate		
	*p*-Value	HR	(95% CI)	*p*-Value	HR	(95% CI)
Pre-transplant Characteristics						
Primary/salvage transplant	0.12					
Cadaveric/living related	0.16					
Whole graft/partial graft	0.16					
AFP > 200 ng/mL upon transplant	0.11					
Explant characteristics						
No. of tumour	0.48					
Size of largest tumour (cm)	0.51					
Degree of differentiation	0.66					
Vascular permeation	0.72					
Within Milan criteria	0.59					
Within UCSF criteria	0.57					
Tumour necrosis	0.64					
Acute rejection < 6 mo post-transplant	0.13					
Recurrence characteristics						
Age at recurrence	0.91					
Early recurrence (<1 year from transplant)	<0.001	2.35	(1.64–3.38)	<0.001 *	2.53	(1.65–3.88)
Date of recurrence	0.01	0.947	(0.910–0.985)	0.89		
Number of recurrences	<0.001	1.01	(1.01–1.02)	0.59		
Size of largest recurrence	0.02	1.11	(1.02–1.20)	0.03 *	1.12	(1.01–1.23)
Number of organs involved	0.01	1.45	(1.11–1.89)	0.03 *	1.48	(1.05–2.07)
Site of recurrence						
Liver	0.01	1.63	1.14–2.33)	0.01 *	1.74	(1.12–2.68)
Lung	0.38					
Bone	0.16					
Peritoneal	0.53					
Adrenal	0.51					
Lymph node	0.48					
AFP ≥ 200 ng/mL upon recurrence	<0.001	2.20	(1.50–3.22)	0.03 *	1.62	(1.04–2.52)
Treatment characteristics						
Immunosuppression						
mTOR inhibitor	<0.001	0.489	(0.341–0.703)	<0.001 *	0.334	(0.202–0.551)
Calcineurin inhibitor	0.07					
Systemic therapy	0.81					
Targeted therapy	0.50					
Chemotherapy	0.45					
Immunotherapy	0.66					
Radical treatment	<0.001	0.317	(0.216–0.468)	<0.001 *	0.327	(0.203–0.528)
Regional treatment	0.42					
Supportive care	<0.001	2.76	(1.66–4.60)	0.60		

AFP: alpha-fetoprotein; HIFU: high intensity focused ultrasound; HR: hazards ratio; PET-CT: positron emission tomography-computed tomography; mTOR: mammalian target of rapamycin; UCSF: University of California San Francisco. * signify statistical significance.

**Table 3:** Characteristics of patients with early versus late recurrence.

	Early Recurrence (n = 71) Median (IQR), *n* (%), or Median ± Standard Error	Late Recurrence (*n* = 73) Median (IQR), *n* (%), or Median ± Standard Error	*p*-Value
Pre-transplant Characteristics			
Age at transplant	55 (45–59)	56 (52–60)	0.19
Gender (M/F) (%M)	64/7 (90%/10%)	69/4 (95%/5%)	0.32
Aetiology			
HBV	65 (92%)	66 (90%)	0.81
HCV	2 (3%)	4 (5%)	0.42
Alcoholic liver disease	1 (1%)	4 (5%)	0.18
Primary/salvage transplant	24/47 (34%/66%)	27/46 (37%/63%)	0.69
Cadaveric/living related	53/18 (75%/25%)	47/26 (64%/36%)	0.18
Whole graft/partial graft	53/18 (75%/25%)	47/26 (64%/36%)	0.18
AFP at time of transplant (ng/mL)	278 (24–5243)	69 (12–404)	0.01 *
Explant characteristics			
No. of tumour	3 (1–9)	2 (1–3)	0.17
Size of largest tumour (cm)	4.0 (2.9–7.1)	4.0 (2.5–6.0)	0.34
Degree of differentiation			0.61
Well	5/25 (20%)	4/36 (11%)	
Moderate	21/25 (84%)	28/36 (78%)	
Poor	3/25 (12%)	4/36 (11%)	
Vascular permeation	18/25 (72%)	16/33 (48%)	0.07
Within Milan criteria	12/59 (20%)	25/65 (38%)	0.03 *
Within UCSF criteria	14/59 (24%)	28/65 (43%)	0.02 *
Tumour necrosis	13/26 (50%)	20/35 (57%)	0.58
Recurrence characteristics			
Age at recurrence	55 (45–60)	58 (53–64)	0.003 *
Time from transplant (months)	6 (4–9)	24 (15–45)	<0.001 *
Number of recurrences	5 (2–9)	2 (1–5)	0.001 *
Size of largest tumour (cm)	1.8 (0.9–3.4)	2.4 (1.2–3.4)	0.07
Number of organs involved	1 (1–2)	1 (1–1)	0.13
Site of recurrence			
Liver	30 (42%)	31 (42%)	0.98
Lung	44 (62%)	28 (38%)	0.01 *
Bone	14 (20%)	9 (12%)	0.23
Peritoneum	3 (4%)	10 (14%)	0.047 *
Adrenal	3 (4%)	9 (12%)	0.08
Lymph node	6 (8%)	4 (5%)	0.48
AFP upon recurrence (ng/mL)	85 (8–961)	7 (3–41)	<0.001 *
Treatment characteristics			
Immunosuppression			
Calcineurin inhibitor	59 (83%)	53 (73%)	0.13
mTOR inhibitor	39 (55%)	40 (55%)	0.99
Radical Therapy	17 (24%)	33 (45%)	0.01 *
Surgical resection	15 (21%)	30 (41%)	0.01 *
Liver	2 (3%)	7 (10%)	0.22
Lung	13 (18%)	14 (19%)	0.58
Adrenal	0 (0%)	6 (8%)	0.01
Bone	0 (0%)	1 (1%)	0.32
Others	1 (1%)	7 (10%)	0.03 *
Ablation	6 (8%)	10 (14%)	0.32
RFA	5 (7%)	10 (14%)	0.19
Alcohol injection	1 (1%)	0 (0%)	0.31
Palliative therapy	41 (58%)	35 (48%)	
Local therapy			
HIFU	2 (3%)	1 (1%)	0.22
SBRT	5 (7%)	12 (16%)	0.18
Regional therapy			
TACE	14 (20%)	16 (22%)	0.75
SIRT	0 (0%)	1 (1%)	0.32
Systemic therapy			
Targeted therapy	31 (44%)	36 (49%)	0.5
Chemotherapy	14 (20%)	11 (15%)	0.46
Immunotherapy	1 (1%)	2 (3%)	0.58
Supportive care	13 (18%)	5 (7%)	0.01 *
Median survival (months)	10.5 ± 1	25.2 ± 3.3	<0.001 *

AFP: alpha-fetoprotein; HBV: hepatitis B virus; HCV: hepatitis C virus; HIFU: high intensity focused ultrasound; PET-CT: positron emission tomography-computed tomography; mTOR: mammalian target of rapamycin; RFA: radiofrequency ablation; SIRT: selective internal radiation therapy. * signify statistical significance.

**Table 4:** Characteristics of patients with high versus low AFP upon recurrence.

	High AFP (*n* = 39) Median (IQR), *n* (%), or Median ± Standard Error	Low AFP (*n* = 105) Median (IQR), *n* (%), or Median ± Standard Error	*p*-Value
Pre-transplant Characteristics			
Age at transplant	53 (44–59)	55 (50–62)	0.07
Gender (M/F) (%M)	36/3 (92%/8%)	97/8 (92%/8%)	0.99
Aetiology			
HBV	35 (90%)	69 (66%)	0.75
HCV	1 (3%)	5 (5%)	0.56
Alcoholic liver disease	0 (0%)	5 (5%)	0.17
Primary/salvage transplant	27/12 (69%/31%)	66/39 (63%/37%)	0.48
Cadaveric/living related	31/8 (79%/21%)	69/36 (66%/34%)	0.11
Whole graft/partial graft	31/8 (79%/21%)	69/36 (66%/34%)	0.11
AFP at time of transplant (ng/mL)	1135 (209–22,004)	56 (11–338)	<0.001 *
Explant characteristics			
No. of tumour	1 (1–4)	2 (1–6)	0.18
Size of largest tumour (cm)	4.0 (2.4–7.6)	4.0 (2.8–6.0)	0.55
Degree of differentiation			0.23
Well	0/10 (0%)	3/51 (6%)	
Moderate	10/10 (100%)	39/61 (64%)	
Poor	0/10 (0%)	7/61 (11%)	
Vascular permeation	5/8 (63%)	29/50 (58%)	0.81
Within Milan criteria	8/32 (25%)	29/92 (32%)	0.49
Within UCSF criteria	9/32 (28%)	33/92 (36%)	0.43
Tumour necrosis	5/9 (56%)	28/52 (54%)	0.92
Recurrence characteristics			
Age at recurrence	56 (45–59)	57 (52–64)	0.02 *
Time from transplant (months)	6 (4–13)	14 (8–29)	0.004 *
Number of recurrences	3 (1–9)	3 (1–7)	0.49
Size of largest tumour (cm)	2.3 (1.1–4.0)	2.0 (1.1–3.3)	0.97
Number of organs involved	1 (1–2)	1 (1–1)	0.04 *
Site of recurrence			
Liver	17 (44%)	44 (42%)	0.86
Lung	21 (54%)	51 (49%)	0.57
Bone	7 (18%)	16 (15%)	0.69
Peritoneum	6 (15%)	7 (7%)	0.11
Adrenal	3 (8%)	9 (9%)	0.87
Lymph node	6 (15%)	4 (4%)	0.02 *
AFP upon recurrence (ng/mL)	1128 (433–6012)	7 (3–32)	<0.001 *
Treatment characteristics			
Immunosuppression			
Calcineurin inhibitor	30 (77%)	82 (78%)	0.88
mTOR inhibitor	18 (46%)	61 (58%)	0.2
Radical Therapy	7 (18%)	43 (41%)	0.01 *
Surgical resection	5 (13%)	40 (38%)	0.004 *
Liver	1 (3%)	8 (8%)	0.52
Lung	3 (8%)	24 (23%)	0.18
Adrenal	0 (0%)	6 (6%)	0.13
Bone	0 (0%)	1 (1%)	0.54
Others	1 (3%)	7 (7%)	0.34
Ablation	2 (5%)	14 (13%)	0.16
RFA	2 (5%)	13 (12%)	0.21
Alcohol injection	0 (0%)	1 (1%)	0.54
Palliative therapy	25 (64%)	51 (49%)	
Local therapy			
HIFU	1 (3%)	2 (2%)	0.64
SBRT	4 (10%)	13 (12%)	0.81
Regional therapy			
TACE	8 (21%)	22 (21%)	0.95
SIRT	0 (0%)	1 (1%)	0.54
Systemic therapy			
Targeted therapy	18 (46%)	49 (47%)	0.96
Chemotherapy	7 (18%)	18 (17%)	0.91
Immunotherapy	1 (3%)	2 (2%)	0.81
Supportive care	7 (18%)	11 (10%)	0.11
Median survival (months)	10.0 ± 1.8	19.5 ± 3	<0.001 *

AFP: alpha-fetoprotein; HBV: hepatitis B virus; HCV: hepatitis C virus; HIFU: high intensity focused ultrasound; PET-CT: positron emission tomography-computed tomography; mTOR: mammalian target of rapamycin; RFA: radiofrequency ablation; SIRT: selective internal radiation therapy. * signify statistical significance.

**Table 5:** Characteristics of patients with and without intrahepatic recurrence.

	Intrahepatic Recurrence (*n* = 61) Median (IQR), *n* (%), or Median ± Standard Error	No Intrahepatic Recurrence (*n* = 83) Median (IQR), *n* (%), or Median ± Standard Error	*p*-Value
Pre-transplant Characteristics			
Age at transplant	55 (48–60)	56 (48–61)	0.86
Gender (M/F) (%M)	57/4 (93%/7%)	76/7 (92%/8%)	0.68
Aetiology			
HBV	58 (95%)	73 (88%)	0.14
HCV	1 (2%)	5 (6%)	0.19
Alcoholic liver disease	2 (3%)	3 (4%)	0.91
Primary/salvage transplant	25/36 (41%/59%)	26/57 (31%/69%)	0.23
Cadaveric/living related	48/13 (79%/21%)	52/31 (63%/37%)	0.04 *
Whole graft/partial graft	48/13 (79%/21%)	52/31 (63%/37%)	0.04 *
AFP at time of transplant (ng/mL)	138 (15–822)	116 (18–929)	0.97
Explant characteristics			
No. of tumour	2 (1–3)	2 (1–7.5)	0.26
Size of largest tumour (cm)	4.0 (2.5–6.8)	4.0 (2.8–6.0)	0.94
Degree of differentiation			0.87
Well	2/18 (11%)	3/43 (7%)	
Moderate	14/18 (78%)	35/43 (81%)	
Poor	2/18 (11%)	5/43 (12%)	
Vascular permeation	10/19 (53%)	24/39 (62%)	0.52
Within Milan criteria	16/53 (30%)	21/71 (30%)	0.94
Within UCSF criteria	18/53 (34%)	24/71 (34%)	0.99
Tumour necrosis	11/19 (58%)	22/42 (52%)	0.69
Recurrence characteristics			
Age at recurrence	57 (50–62)	57 (49–63)	0.99
Time from transplant (months)	12 (6–21)	12 (6–27)	0.57
Number of recurrences	4 (2–9)	3 (1–6)	0.11
Size of largest tumour (cm)	2.7 (1.9–3.5)	1.5 (0.9–2.9)	0.04 *
Number of organs involved	1 (1–2)	1 (1–1)	<0.001 *
Site of recurrence			
Liver	61 (100%)	0 (0%)	<0.001 *
Lung	18 (30%)	54 (65%)	<0.001 *
Bone	7 (11%)	16 (19%)	0.21
Peritoneum	2 (3%)	11 (13%)	0.04
Adrenal	4 (7%)	8 (10%)	0.51
Lymph node	6 (10%)	4 (5%)	0.24
AFP upon recurrence (ng/mL)	27 (4–444)	16 (4–203)	0.65
Treatment characteristics			
Immunosuppression			
Calcineurin inhibitor	48 (79%)	64 (77%)	0.82
mTOR inhibitor	34 (56%)	45 (54%)	0.86
Radical Therapy	19 (31%)	31 (37%)	0.44
Surgical resection	14 (23%)	31 (37%)	0.07
Liver	9 (15%)	0 (0%)	0.01 *
Lung	6 (10%)	21 (25%)	0.11
Adrenal	3 (5%)	3 (4%)	0.7
Bone	0 (0%)	1 (1%)	0.39
Others	0 (0%)	8 (10%)	0.01 *
Ablation	11 (18%)	5 (6%)	0.02 *
RFA	10 (16%)	5 (6%)	0.04
Alcohol injection	1 (2%)	0 (0%)	0.24
Palliative therapy	31 (51%)	45 (54%)	
Local therapy			
HIFU	2 (3%)	1 (1%)	0.49
SBRT	7 (11%)	10 (12%)	0.47
Regional therapy			
TACE	30 (49%)	0 (0%)	
SIRT	1 (2%)	0 (0%)	0.39
Systemic therapy			
Targeted therapy	25 (41%)	42 (51%)	0.25
Chemotherapy	8 (13%)	17 (20%)	0.25
Immunotherapy	0 (0%)	2 (2%)	0.75
Supportive care	11 (18%)	7 (8%)	0.53
Median survival (months)	13.7 ± 2.2	18.8 ± 2.3	0.01 *

AFP: alpha-fetoprotein; HBV: Hepatitis B virus; HCV: hepatitis C virus; HIFU: high intensity focused ultrasound; PET-CT: positron emission tomography-computed tomography; mTOR: mammalian target of rapamycin; RFA: radiofrequency ablation; SIRT: selective internal radiation therapy. * signify statistical significance.

**Table 6:** Survival outcomes of radical versus palliative treatment stratified by biological risk factors.

	Overall (*n* = 144)		Radical (*n* = 50)		Palliative (*n* = 76)		*p*-Value	Survival Gain
	No.	Survival (Months)	No.	Survival (Months)	No.	Survival (Months)		(Months)
Recurrence ≥ 1 year	73	25.2 ± 3.3	33	31.5 ± 2.2	35	17.9 ± 2.7	<0.001 *	13.6
Recurrence < 1 year	71	10.5 ± 1.0	17	18.2 ± 1.5	41	9.2 ± 1.5	<0.001 *	9.0
No liver recurrence	83	18.8 ± 2.3	31	31.5 ± 5.5	45	14.6 ± 3.3	<0.001 *	16.9
Liver recurrence	61	13.7 ± 2.2	19	28.0 ± 4.5	31	11.6 ± 2.0	<0.001 *	16.4
AFP < 200	105	19.5 ± 3.0	43	31.0 ± 1.0	51	15.6 ± 2.7	<0.001 *	15.4
AFP ≥ 200	39	10.0 ± 1.8	7	18.2 ± 6.5	25	8.8 ± 2.2	0.13	9.3
No. of risk factors								
0	36	31.0 ± 3.8	18	55.5 ± 18.5	17	26.5 ± 5.0	0.02 *	29.0
1	58	16.7 ± 3.0	23	30.2 ± 3.0	27	10.5 ± 1.7	<0.001 *	19.7
2–3	50	9.8 ± 2.8	9	13.2 ± 0.4	32	9.8 ± 2.3	0.047 *	3.4

AFP: alpha-fetoprotein. * signify statistical significance.

**Table 7:** Reported biological risk factors affecting post-recurrence survival.

	Pre-Transplant	Early Rejection	Early Recurrence	AFP on Recurrence	Site of Recurrence
Toso et al. [26]	-	<6 months	Time to recur	-	-
Roayaie et al. [23]	-	-	Time to recur	-	Bone
Sapisochin et al. [27]	-	-	<1 year	>100 ng/mL	-
Bodzin et al. [24]	Neutrophil-lymphocyte ratio	-	Time to recur	AFP level	Bone
Au et al. (current study)	-	-	<1 year	>200 ng/mL	Liver

AFP: alpha-fetoprotein.

## Data Availability

The data presented in this study are available on request from the corresponding author. The data are not publicly available due to patient privacy.

## Supplementary Materials

The following supporting information can be downloaded at: https://www.mdpi.com/article/10.3390/jcm11154389/s1, Table S1: Characteristics of all patients with post-transplant HCC recurrence; Table S2: Characteristics of radical treatment.

## References

[1] Sapisochin G., Bruix J. (2017). Liver transplantation for hepatocellular carcinoma: Outcomes and novel surgical approaches. Nat. Rev. Gastroenterol. Hepatol..

[2] Pinna A.D., Yang T., Mazzaferro V., de Carlis L., Zhou J., Roayaie S., Cucchetti A. (2018). Liver Transplantation and Hepatic Resection can Achieve Cure for Hepatocellular Carcinoma. Ann. Surg..

[3] Kwong A., Kim W.R., Lake J.R., Smith J.M., Schladt D.P., Skeans M.A., Kasiske B.L. (2020). OPTN/SRTR 2018 Annual Data Report: Liver. Am. J. Transplant..

[4] Adam R., Karam V., Delvart V., O’Grady J., Mirza D., Klempnauer J., Burroughs A. (2012). Evolution of indications and results of liver transplantation in Europe. A report from the European Liver Transplant Registry (ELTR). J. Hepatol..

[5] Yang J.D., Larson J., Watt K.D., Allen A.M., Wiesner R.H., Gores G.J., Leise M.D. (2017). Hepatocellular Carcinoma Is the Most Common Indication for Liver Transplantation and Placement on the Waitlist in the United States. Clin. Gastroenterol. Hepatol. Off. Clin. Pract. J. Am. Gastroenterol. Assoc..

[6] Mazzaferro V., Regalia E., Doci R., Andreola S., Pulvirenti A., Bozzetti F., Gennari L. (1996). Liver transplantation for the treatment of small hepatocellular carcinomas in patients with cirrhosis. N. Engl. J. Med..

[7] Yao F.Y., Ferrell L., Bass N.M., Watson J.J., Bacchetti P., Venook A., Roberts J.P. (2001). Liver transplantation for hepatocellular carcinoma: Expansion of the tumor size limits does not adversely impact survival. Hepatology.

[8] Centonze L., di Sandro S., Lauterio A., de Carlis R., Sgrazzutti C., Ciulli C., De Carlis L. (2021). A retrospective single-centre analysis of the oncological impact of LI-RADS classification applied to Metroticket 2.0 calculator in liver transplantation: Every nodule matters. Transpl. Int..

[9] Ivanics T., Nelson W., Patel M.S., Claasen M.P., Lau L., Gorgen A., Sapisochin G. (2022). The Toronto Postliver Transplantation Hepatocellular Carcinoma Recurrence Calculator: A Machine Learning Approach. Liver Transplant..

[10] Mazzaferro V., Sposito C., Zhou J., Pinna A.D., de Carlis L., Fan J., Cucchetti A. (2018). Metroticket 2.0 Model for Analysis of Competing Risks of Death After Liver Transplantation for Hepatocellular Carcinoma. Gastroenterology.

[11] De’angelis N., Landi F., Azoulay D., Carra M.C. (1118). Managements of recurrent hepatocellular carcinoma after liver transplantation: A systematic review. World J. Gastroenterol..

[12] Filgueira N.A. (2019). Hepatocellular carcinoma recurrence after liver transplantation: Risk factors, screening and clinical presentation. World J. Hepatol..

[13] Schnitzbauer A.A., Zuelke C., Graeb C., Rochon J., Bilbao I., Burra P., Geissler E.K. (2010). A prospective randomised, open-labeled, trial comparing sirolimus-containing versus mTOR-inhibitor-free immunosuppression in patients undergoing liver transplantation for hepatocellular carcinoma. BMC Cancer.

[14] Schnitzbauer A.A., Schlitt H.J., Geissler E.K. (2011). Influence of immunosuppressive drugs on the recurrence of hepatocellular carcinoma after liver transplantation: A gap between basic science and clinical evidence. Transplantation.

[15] Au K.P., Chok K.S.H. (2020). Mammalian target of rapamycin inhibitors after post-transplant hepatocellular carcinoma recurrence: Is it too late?. World J. Gastrointest. Surg..

[16] Fallah-Rad N., Cao Y., Knox J.J., Jang R.W.-J., Dhani N.C., Sapisochin G., Chen E. (2017). Sorafenib treatment in recurrent hepatocellular carcinoma post liver transplantation. J. Clin. Oncol..

[17] Niibe Y., Hayakawa K. (2010). Oligometastases and oligo-recurrence: The new era of cancer therapy. Jpn. J. Clin. Oncol..

[18] Au K.P., Fung J.Y.Y., Dai W.C., Chan A.C.Y., Lo C.M., Chok K.S.H. (2022). Verifying the Benefits of Radical Treatment in Post-Transplant Hepatocellular Carcinoma Oligo-recurrence: A Propensity Score Analysis. Liver Transplant..

[19] Testa G., Goldstein R.M., Toughanipour A., Abbasoglu O., Jeyarajah R., Levy M.F., Klintmalm G.B. (1998). Guidelines for surgical procedures after liver transplantation. Ann. Surg..

[20] Marangoni G., Faraj W., Sethi H., Rela M., Muiesan P., Heaton N. (2008). Liver resection in liver transplant recipients. Hepatobiliary Pancreat. Dis. Int..

[21] Sommacale D., Dondero F., Sauvanet A., Francoz C., Durand F., Farges O., Belghiti J. (2013). Liver Resection in Transplanted Patients: A Single-Center Western Experience. Transplant. Proc..

[22] Chok K.S. (2015). Management of recurrent hepatocellular carcinoma after liver transplant. World J. Hepatol..

[23] Roayaie S., Schwartz J.D., Sung M.W., Emre S.H., Miller C.M., Gondolesi G.E., Schwartz M.E. (2004). Recurrence of hepatocellular carcinoma after liver transplant: Patterns and prognosis. Liver Transplant..

[24] Bodzin A.S., Lunsford K.E., Markovic D., Harlander-Locke M.P., Busuttil R.W., Agopian V.G. (2017). Predicting Mortality in Patients Developing Recurrent Hepatocellular Carcinoma After Liver Transplantation. Ann. Surg..

[25] Au K.P., Chok K.S.H. (2018). Multidisciplinary approach for post-liver transplant recurrence of hepatocellular carcinoma: A proposed management algorithm. World J. Gastroenterol..

[26] Toso C., Mentha G., Majno P. (2013). Integrating sorafenib into an algorithm for the management of post-transplant hepatocellular carcinoma recurrence. J. Hepatol..

[27] Sapisochin G., Goldaracena N., Astete S., Laurence J.M., Davidson D., Rafael E., Greig P.D. (2015). Benefit of Treating Hepatocellular Carcinoma Recurrence after Liver Transplantation and Analysis of Prognostic Factors for Survival in a Large Euro-American Series. Ann. Surg. Oncol..

[28] Poon R.T. (2009). Differentiating early and late recurrences after resection of HCC in cirrhotic patients: Implications on surveillance, prevention, and treatment strategies. Ann. Surg. Oncol..

[29] Du Z.G., Wei Y.G., Chen K.F., Li B. (2014). Risk factors associated with early and late recurrence after curative resection of hepatocellular carcinoma: A single institution’s experience with 398 consecutive patients. Hepatobiliary Pancreat. Dis. Int..

[30] Chok K.S.H., Chan S.C., Cheung T.T., Chan A.C.Y., Fan S.T., Lo C.M. (2011). Late Recurrence of Hepatocellular Carcinoma after Liver Transplantation. World J. Surg..

[31] Wu J.W., Huang Y.H., Chau G.Y., Su C.W., Lai C.R., Lee P.C., Lui W.Y. (2009). Risk factors for early and late recurrence in hepatitis B-related hepatocellular carcinoma. J. Hepatol..

[32] Mazzaferro V., Llovet J.M., Miceli R., Bhoori S., Schiavo M., Mariani L., Metroticket Investigator Study Group (2009). Predicting survival after liver transplantation in patients with hepatocellular carcinoma beyond the Milan criteria: A retrospective, exploratory analysis. Lancet Oncol..

