# Supplementary material for: Impact of Tumour Biology on Outcomes of Radical Therapy for Hepatocellular Carcinoma Oligo-Recurrence after Liver Transplantation
Source: J Clin Med. 2022 Jul 28;11(15):4389. doi: 10.3390/jcm11154389 (PMC9368948; doi:10.3390/jcm11154389)
Supplement: Supplementary file 1 [file jcm-11-04389-s001.zip › Supple Table S1.pdf]

Supplementary Table S1. Characteristics of all patients with post-transplant HCC recurrence (n=144).

Pre-transplant Characteristics								
Age at transplant	55			(	48	-	60	)
Gender (M/F) (%M)	133	/	11	(	92%	/	8%	)
Aetiology								
HBV	131			(	91%			)
HCV	6			(	4%			)
Alcoholic liver disease	5			(	3%			)
Pre-operative tumour treatment	77			(	53%			)
Resection	34			(	24%			)
RFA	25			(	17%			)
TACE	52			(	36%			)
Systemic therapy	6			(	4%			)
Waitlist time (month) *	1.3			(	0.5	-	5.0	)
Primary / salvage transplant	93	/	51	(	65%	/	35%	)
Cadaveric / living related	100	/	44	(	69%	/	31%	)
Whole graft / partial graft	100	/	44	(	69%	/	31%	)
AFP at time of transplant (ng/ml)	122			(	18	-	846	)
Explant characteristics								
No. of tumour	2			(	1	-	5	)
Size of largest tumour (cm)	4.0			(	2.7	-	6.5	)
Degree of differentiation								
Well	5	/	61	(	8%			)
Moderate	49	/	61	(	80%			)
Poor	7	/	61	(	11%			)
Vascular permeation	34	/	58	(	59%			)
Within Milan criteria	36	/	124	(	29%			)
Within UCSF criteria	42	/	124	(	34%			)
Tumour necrosis	33	/	61	(	54%			)
Recurrence characteristics								
Age at recurrence	57			(	50	-	63	)
Time from transplant (months)	12			(	6	-	25	)
Date of recurrence	11/2009			(	8/2006	-	5/2015	)
Staging PET-CT	57			(	40%			)
Number of recurrences	3			(	1	-	8	)
Size of largest tumour (cm)	2.0			(	1.1	-	3.4	)
Number of organs involved	1			(	1	-	1	)
Site of recurrence								
Liver	61			(	42%			)
Lung	72			(	50%			)
Bone	23			(	16%			)
Peritoneum	13			(	9%			)
Adrenal	12			(	8%			)
Lymph node	10			(	7%			)
AFP upon recurrence (ng/ml)	17			(	4	-	229	)

Treatment characteristics				
Immunosuppression				
Calcineurin inhibitor	112	(	78%	)
mTOR inhibitor	79	(	55%	)
Curative Therapy	50	(	35%	)
Surgical resection	45	(	31%	)
Liver	9	(	6%	)
Lung	27	(	19%	)
Adrenal	6	(	4%	)
Bone	1	(	1%	)
Others	8	(	6%	)
Ablation	16	(	11%	)
RFA	15	(	10%	)
Alcohol injection	1	(	1%	)
Palliative therapy	76	(	53%	)
Local therapy				
HIFU	3	(	2%	)
SBRT	17	(	12%	)
Regional therapy				
TACE	30	(	21%	)
SIRT	1	(	1%	)
Systemic therapy	75	(	52%	)
Targeted therapy	67	(	47%	)
Chemotherapy	25	(	17%	)
Immunotherapy	3	(	2%	)
Supportive care	18	(	13%	)
Median survival (months)		16.1 ± 1.1		

*For patients transplanted in our centre

AFP: alpha-fetoprotein; HBV: Hepatitis B virus; HCV: hepatitis C virus;
HIFU: high intensity focused ultrasound; PET-CT: positron emission tomography-computed tomography;
mTOR: mammalian target of rapamycin; RFA: radiofrequency ablation; SBRT: stereotactic body radiotherapy;
SIRT: selective internal radiation therapy; TACE: trans-arterial chemoembolization;
UCSF: University of California San Francisco
