# Supplementary material for: Impact of Tumour Biology on Outcomes of Radical Therapy for Hepatocellular Carcinoma Oligo-Recurrence after Liver Transplantation
Source: J Clin Med. 2022 Jul 28;11(15):4389. doi: 10.3390/jcm11154389 (PMC9368948; doi:10.3390/jcm11154389)
Supplement: Supplementary file 1 [file jcm-11-04389-s001.zip › Supple Table S2.pdf]

Supplementary Table S2. Characteristics of radical treatment (total courses of treatment =76).

Treatment	Number
Surgical resection	60 (79%)
Liver	10 (13%)
Wedge resection	5 (7%)
Segmentectomy	2 (3%)
Left lateral sectionectomy	2 (3%)
Right hepatectomy	1 (1%)
Lung	36 (47%)
Wedge resection	33 (43%)
Lobectomy	3 (4%)
Adrenalectomy	6 (8%)
Peritoneal metastasis	4 (5%)
Others	4 (5%)
Ablation	16 (21%)
RFA	15 (20%)
Alcohol injection	1 (1%)

RFA: radiofrequency ablation
